# Molecular Pathology of Pancreatic Ductal Adenocarcinoma

**DOI:** 10.3390/cancers17213549

**Published:** 2025-11-02

**Authors:** Akram Shalaby, Navid Sadri, Yue Xue

**Affiliations:** Department of Pathology, University Hospitals Cleveland Medical Center, Case Western Reserve University School of Medicine, Cleveland, OH 44106, USA; akram.shalaby@uhhospitals.org (A.S.); navid.sadri@uhhospitals.org (N.S.)

**Keywords:** pancreatic ductal adenocarcinoma, histologic variant, pancreatic intraductal neoplasia, intraductal papillary mucinous neoplasm, intraductal oncocytic papillary neoplasm, mucinous cystic neoplasm, intraductal tubulopapillary neoplasm, molecular pathology

## Abstract

**Simple Summary:**

Pancreatic ductal adenocarcinoma (PDAC) is considered one of the deadliest types of cancers, often detected at advanced stages and showing limited response to conventional therapies. Its marked molecular heterogeneity continues to pose major challenges for both clinicians and researchers. Understanding the genetic and molecular underpinnings of PDAC is essential for elucidating the mechanisms of tumor initiation and progression, thereby paving the way for the development of effective targeted therapies. In this paper, we review the genetic alterations underlying PDAC and its precursor lesions, with an emphasis on the therapeutic implications of these molecular pathways. We also discuss the current literature on molecular subtypes of PDAC and their impact on clinical outcomes.

**Abstract:**

Pancreatic ductal adenocarcinoma (PDAC) is an aggressive cancer that frequently presents at an advanced stage with limited effective treatment options and a dismal prognosis. It is a highly heterogenous disease driven by various genetic and epigenetic alterations. Recent advances in sequencing modalities have significantly improved our understanding of the genetics of PDAC, which could lead to promising and novel therapeutic strategies. In this review, we summarize the most up-to-date literature on the molecular landscape of PDAC and its precursor lesions, as well as recent advances in targeted therapy.

## 1. Introduction

Pancreatic ductal adenocarcinoma (PDAC) is an aggressive malignancy originating from the ductal epithelial cells of the exocrine pancreas. It ranks among the deadliest forms of cancer and is projected to become the second leading cause of cancer-related mortality by 2030 [1,2]. Due to the absence of specific clinical symptoms and the lack of reliable diagnostic biomarkers for early detection, the majority of patients are diagnosed at an advanced stage, often with locally advanced or metastatic disease that is not amenable to surgical resection. Compounding this challenge, PDAC is commonly resistant to most currently available therapeutic agents. In response, considerable research efforts have focused on elucidating the molecular mechanisms driving PDAC, with the goal of developing effective, targeted therapies. In this review, we highlight key genetic studies that have shaped our current understanding of PDAC pathogenesis.

## 2. Genetic Alterations in PDAC

Both conventional studies and more recent next-generation sequencing (NGS) analyses have revealed that PDAC exhibits a highly heterogeneous molecular landscape. Among the most frequently altered genes are *KRAS* (Kirsten rat sarcoma viral oncogene), *TP53* (tumor suppressor gene), *SMAD4* (also known as Deleted in Pancreatic Cancer-4, *DPC4*), and *CDKN2A* (cyclin-dependent kinase inhibitor 2A) [3].

Activating mutations in *KRAS*, particularly at codons 12, 13, or 61, are found in over 90% of PDACs, with *KRAS^G12D^* and *KRAS^G12V^* accounting for 39.2% and 32.5% of all *KRAS* mutations, respectively [4]. *KRAS* encodes a small GTPase protein that functions as a molecular switch in signal transduction. When mutated, it constitutively activates several downstream signaling pathways—including the RAF–mitogen-activated protein kinase (MAPK), Ral guanine nucleotide dissociation stimulator (RalGDS), and phosphoinositide 3-kinase (PI3K) pathways—driving key oncogenic processes such as cancer cell proliferation, apoptosis resistance, migration, and metastasis [3]. *KRAS* mutations are also detected in early precursor lesions of PDAC, including low-grade pancreatic intraepithelial neoplasia (PanINs) and intraductal papillary mucinous neoplasms (IPMNs), underscoring their role in the initiation of neoplastic transformation [5]. Inactivating mutations in *TP53* are present in approximately 50% to 80% of PDAC cases [6,7,8]. The *TP53* gene encodes the tumor suppressor protein p53, a key regulator of cellular responses to genotoxic stress. p53 maintains genomic stability by inducing cell cycle arrest, DNA repair, or apoptosis in response to damage [9]. Loss of p53 function contributes to tumor progression and has also been implicated in promoting epithelial-to-mesenchymal transition (EMT) via upregulation of ZEB1, a zinc-finger transcription factor that suppresses epithelial gene expression and enhances cell motility, migration, and metastatic potential [10].The tumor suppressor gene *CDKN2A* (also known as *p16^INK4a^*) is altered in approximately 95% of PDACs through mechanisms such as homozygous deletion, intragenic mutation, or promoter hypermethylation [11,12]. p16 inhibits CDK4/6-mediated phosphorylation of the retinoblastoma (RB) protein, thereby regulating cell cycle progression and preventing premature entry into the S phase. Inactivation of p16 disrupts this control, accelerating cellular proliferation [13]. Germline truncating mutations in *CDKN2A*, such as *E119X* and *Q50X*, have recently been identified in patients with familial “pancreatic cancer plus melanoma syndrome”, a rare cancer predisposition syndrome associated with increased risk of pancreatic cancer and malignant melanoma [14]. Inactivating mutations or deletions in *SMAD4* (also known as *DPC4*), another key tumor suppressor gene, are observed in 30% to 60% of PDACs [3,6,15]. *SMAD4* encodes a central mediator of the transforming growth factor-beta (TGF-β) signaling pathway, which normally functions to inhibit cell proliferation and maintain tissue homeostasis. Disruption of this pathway results in unchecked cellular growth and contributes to tumor progression [16]. Similar to *KRAS*, loss-of-function alterations in *TP53* and *SMAD4* are also observed in PDAC precursor lesions, though they typically emerge later during the progression to high-grade dysplasia [17].

In a recent study, Campbell et al. analyzed 508 patients with resected PDAC for mutations *in KRAS*, *TP53*, *CDKN2A*, and *SMAD4* and their associations with pathological features and overall survival [18]. *KRAS* mutations (89.8%) and *TP53* mutations (57.5%) were linked to more aggressive pathology and shorter median overall survival (mOS), while wild-type *KRAS* or *TP53* correlated with better outcomes. *CDKN2A* (17.3%) and *SMAD4* (20.3%) mutations showed no significant impact on survival. Notably, the presence of two or more driver mutations predicted worse prognosis, highlighting the cumulative effect of genetic alterations. Complementing this, Liviu Badea et al. [19] identified overexpression of genes such as keratin 7, laminin gamma 2, stratifin, platelet phosphofructokinase, annexin A2, *MAP4K4*, and *OACT2* (*MBOAT2*) as associated with poor survival, underscoring the complex molecular landscape influencing PDAC outcomes.

In addition to the commonly mutated driver genes (*KRAS*, *TP53*, *CDKN2A*, and *SMAD4*), a variety of less frequent genetic alterations have been identified in PDAC. These include gene amplifications involving *CMYC* (chromosome 8q), *MYB* (chromosome 6q), *AIB1/NCOA3* (chromosome 20q), *EGFR* (chromosome 7p), and *GATA6* [20]. Germline mutations in genes associated with the Fanconi anemia (FA) DNA repair pathway—such as *BRCA1*, *BRCA2*, and *PALB2*—impair the cellular response to DNA damage, leading to the accumulation of genomic instability. These mutations are present in approximately 5–7% of patients with PDAC [21]. *ATM* germline mutations, which also disrupt DNA damage response, represent the second most common inherited alteration in PDAC and are found in about 6% of cases [22]. Alterations in chromatin-remodeling genes, particularly those encoding components of the SWI/SNF (switch/sucrose non-fermentable) complex, have also been implicated in PDAC [23]. For example, *ARID1A* (AT-rich interaction domain 1A) is a chromatin regulatory protein involved in DNA repair and cell cycle regulation. Loss-of-function mutations in *ARID1A* have been shown to promote pancreatic tumorigenesis through activation of multiple downstream oncogenic pathways [24]. Recent genomic studies have also identified mutations in genes involved in RNA splicing. Mutations in *SF3B1*, for instance, disrupt spliceosome recognition of pre-mRNA, contributing to abnormal splicing patterns [25]. These defects may play a role in tumor initiation and progression by promoting more aggressive disease phenotypes and facilitating metastasis [26].

Several histologic variants of PDAC are associated with specific genetic alterations. Colloid carcinoma, which often arises from intestinal-type IPMNs, is frequently driven by *GNAS* mutations [27]. Adenosquamous carcinoma, a rare and aggressive variant of PDAC, commonly harbors somatic mutations in *UPF1* [28], a gene involved in nonsense-mediated mRNA decay (NMD)—a cellular mechanism that eliminates mRNAs with premature stop codons. Dysfunction of *UPF1* may allow the accumulation of aberrant, toxic transcripts, potentially contributing to tumorigenesis [29]. However, the significance of *UPF1* mutations in adenosquamous carcinoma remains controversial; for instance, a study by Polaski et al. found no significant impact of *UPF1* mutations on tumor growth or squamous differentiation in human and murine models [30]. This subtype may also exhibit canonical mutations in *KRAS*, *TP53*, *CDKN2A/p16*, and *SMAD4*, as well as amplification of the *MYC* oncogene [31,32]. Medullary carcinoma, another rare PDAC subtype, may display microsatellite instability (MSI), similar to medullary colorectal cancers. It is typically associated with a lower frequency of *KRAS* mutations [33] and can occur in individuals with Lynch syndrome or germline mutations in mismatch repair (MMR) genes [34].

As most patients with resectable PDAC receive neoadjuvant therapy before surgery, endoscopic ultrasound-guided fine-needle aspiration (EUS-FNA) or fine-needle biopsy (FNB) specimens are often the only tissue available for molecular testing. A study comparing cytologic and surgical samples demonstrated highly concordant mutational profiles, indicating that cytology specimens are reliable for NGS and can serve as effective alternatives to surgical samples [35]. In addition, Gan et al. reported that both EUS-FNA and EUS-FNB yield sufficient material for targeted NGS analysis, supporting the adequacy of either approach for molecular testing [36]. EUS techniques, however, differ in their efficiency of tissue procurement. In a cohort of 210 patients (146 pancreatic and 64 nonpancreatic lesions), the wet-suction technique achieved a higher tissue core yield than the slow-pull method, although both demonstrated comparable diagnostic accuracy and tumor adequacy [37]. Another study further reported that the modified wet-suction technique yielded superior tissue integrity and sample adequacy compared with the dry-suction and slow-pull methods, whereas the no-suction approach performed significantly worse [38].

## 3. Genetic Alterations in Precursor Lesions of PDAC

Several non-invasive pancreatic precursor lesions are known to give rise to PDAC, including pancreatic intraepithelial neoplasia (PanIN), IPMN, intraductal oncocytic papillary neoplasm (IOPN), mucinous cystic neoplasm (MCN), and intraductal tubulopapillary neoplasm (ITPN).

Feldmann et al. proposed a chronological classification of molecular alterations in PanINs into early, intermediate, and late events. *KRAS* mutations, telomere shortening, and *p21* upregulation were observed across all PanIN grades, indicating their role as early events in tumorigenesis. In contrast, alterations in *TP53*, *SMAD4*, and *BRCA2* were predominantly associated with high-grade PanINs, suggesting they represent later events in the progression toward invasive PDAC [39].

Recent advances in molecular and sequencing technologies have greatly expanded our understanding of the molecular biology of IPMNs. Somatic *KRAS* mutations are among the earliest genetic alterations in IPMN pathogenesis, occurring in approximately 60–80% of cases [27]. These mutations are most commonly associated with the pancreatobiliary subtype and are least frequent in the intestinal subtype [40]. IPMNs also frequently harbor somatic mutations in *GNAS*, an oncogene located on chromosome 20q that encodes the stimulatory alpha subunit of the G-protein (Gαs). Mutations at codon 201 of *GNAS* are identified in about 60% of IPMNs and are more prevalent in higher-grade lesions [40]. In contrast to *KRAS*, *GNAS* mutations are most commonly observed in the intestinal subtype of IPMN. Another gene frequently altered in IPMNs is *RNF43*, which encodes an E3 ubiquitin ligase that negatively regulates the Wnt signaling pathway by promoting the ubiquitination and degradation of Frizzled family Wnt receptors. Somatic mutations in *RNF43* are found in approximately 24% of IPMNs [41,42]. Later-stage genetic alterations, such as inactivating mutations in *TP53* and *SMAD4*, are typically associated with high-grade dysplasia and the transition to invasive carcinoma [43].

IOPN is a recently characterized, distinct subtype of pancreatic intraductal neoplasms. Unlike IPMNs, IOPNs typically lack the common mutations found in IPMNs but instead exhibit recurrent somatic alterations in genes such as *ARHGAP26*, *ASXL1*, *EPHA8*, and *ERBB4* [44]. Notably, a subset of IOPNs has been found to harbor *DNAJB1-PRKACA* gene fusions, a genetic alteration originally identified in fibrolamellar hepatocellular carcinoma [45].

Activating mutations in codon 12 of *KRAS* are detected in approximately 50% of MCNs [46]. Mutations in *RNF43* are also frequently observed in MCNs, particularly in high-grade and invasive lesions, occurring in 56% of such cases compared to 33% in low-grade lesions [47]. Unlike IPMNs, *GNAS* mutations have not been reported in MCNs [48,49]. Additionally, alterations in *CDKN2A*, *TP53*, and *SMAD4* are commonly associated with MCNs exhibiting high-grade dysplasia [50,51].

**Figure 1:**
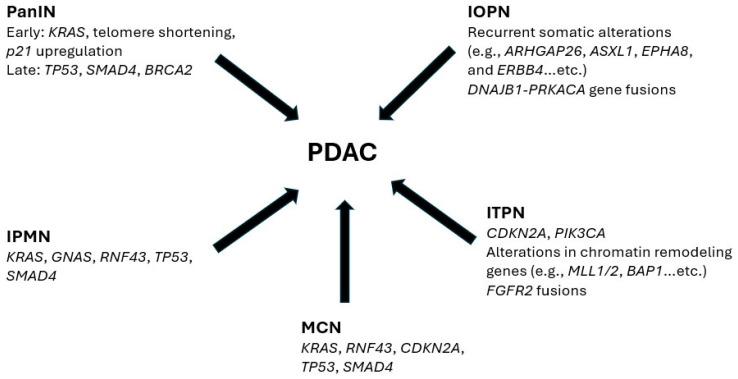
The common genetic alterations found in PDAC and its precursor lesions. IOPN: Intraductal oncocytic papillary neoplasm; IPMN: Intraductal papillary mucinous neoplasm; ITPN: Intraductal tubulopapillary neoplasm; MCN: Mucinous cystic neoplasm; PanIN: Pancreatic intraepithelial neoplasia; PDAC: Pancreatic ductal adenocarcinoma.

ITPN is molecularly distinct from other pancreatic precursor lesions, with *KRAS* and *TP53* mutations being notably rare [52]. In a study by Basturk et al. analyzing 22 ITPN cases through targeted next-generation or whole-exome sequencing, approximately 25% of tumors harbored *CDKN2A* mutations. Mutations in chromatin remodeling genes—including *MLL1*, *MLL2*, *MLL3*, *BAP1*, *PBRM1*, *EED*, and *ATRX*—were identified in about one-third of cases. Additionally, 27% of ITPNs carried mutations in the PI3K pathway, such as *PIK3CA* [53]. Notably, around 20% of ITPNs harbor *FGFR2* gene fusions with various fusion partners, representing a promising therapeutic target. FGFR2 inhibitors like pemigatinib and infigratinib have already been FDA-approved for treating intrahepatic cholangiocarcinoma, highlighting potential treatment avenues for ITPN [54]. Figure 1 summarizes the most common genetic alterations seen in PDAC and its precursor lesions.

## 4. Transcriptomic PDAC Subtypes

PDAC has recently been the focus of intensive transcriptomic analysis, leading to important insights into its molecular subtypes. A landmark study by Collisson et al. was the first to classify PDAC based on transcriptomic data derived from patient-derived cell lines. Their analysis identified three distinct subtypes: classical, quasi-mesenchymal (QM), and exocrine-like—each with unique gene expression profiles, clinical outcomes, and therapeutic sensitivities. The classical subtype exhibited high expression of epithelial and adhesion-associated genes, was linked to the best overall survival, and showed predicted sensitivity to erlotinib, an EGFR inhibitor [55]. In contrast, the QM subtype was characterized by elevated expression of mesenchymal genes, associated with the poorest prognosis, but showed greater sensitivity to gemcitabine, a standard chemotherapeutic agent. The exocrine-like subtype displayed the highest expression of genes involved in digestive enzyme production, although its clinical relevance has been debated [55].

By computationally removing the transcriptional signals contributed by stromal and immune cells from bulk RNA-seq data, Moffitt et al. identified two tumor-specific subtypes of PDAC: classical and basal-like. The classical subtype closely resembled the classical group previously described by Collisson et al., while the basal-like subtype shared molecular features with basal-like breast carcinoma and was associated with a poorer prognosis [56]. In addition to tumor subtypes, Moffitt et al. also defined two distinct stromal subtypes: normal stroma and activated stroma. The normal stroma was enriched for pancreatic stellate cell markers, such as desmin, smooth muscle actin, and vimentin. In contrast, the activated stroma showed high expression of macrophage-associated genes (*ITGAM*, *CCL13*, *CCL18*) and other genes linked to tumor progression, including *SPARC*, *WNT2*, *WNT5A*, *MMP9*, and *MMP11* [56]. Importantly, the combination of tumor and stromal subtypes had prognostic significance. Patients with the classical tumor subtype and activated stroma had significantly worse survival compared to those with classical tumors and normal stroma. However, in tumors of the basal-like subtype, stromal classification did not significantly impact survival, suggesting that tumor-intrinsic features play a dominant role in determining prognosis in these cases [56].

Bailey et al. performed integrated genomic analysis of 456 PDACs and defined four distinct molecular subtypes: squamous, pancreatic progenitor, aberrantly differentiated endocrine exocrine (ADEX), and immunogenic [57]. The squamous subtype (31%) was characterized by genetic alterations in the gene networks responsible for regulating inflammation, hypoxia response, metabolic functions, and TGF-β signaling. It showed upregulation of TP63ΔN and its target genes and was associated with mutations in *TP53* and *KDM6A*. This subtype was associated with the poorest survival. The pancreatic progenitor type (19%) was defined by altered expression of transcription factors that are essential for early pancreatic development, such as PDX1, MNX1, and FOXA2/3. It showed enrichment for genetic pathways regulating fatty acid oxidation, steroid synthesis, and drug metabolism. The ADEX subtype (21%) is considered a subclass of the progenitor group and shows upregulation of genetic pathways related to *KRAS* activation, as well as other genes involved in exocrine differentiation (*NR5A2*, *MIST1*, *RBPJL*) and endocrine differentiation (*NEUROD1*, *INS*, *NKX2-2*, and MODY-related genes). The immunogenic subtype (29%) is characterized by significant immune cell infiltration, particularly CD4+ and CD8+ cells and upregulation of *CTLA4* and *PD1* tumor immune suppression and Toll-like receptor signaling pathways [57].

These molecular subtypes also correlated with distinct histopathological features. The squamous subtype was often associated with adenosquamous carcinoma; the progenitor and immunogenic subtypes were linked to colloid carcinomas and carcinomas arising from IPMNs; and the ADEX subtype was aligned with rare acinar cell carcinomas [57].

Puleo et al. validated the basal-like and classical tumor subtypes previously identified by Moffitt et al. and further expanded upon this classification by incorporating gene expression patterns from the tumor microenvironment. This allowed them to define five distinct PDAC subtypes: pure basal-like, stroma-activated, desmoplastic, pure classical, and immune classical [58]. These subtypes showed strong correspondence with most of the molecular categories proposed by Bailey et al., with one notable exception: Bailey’s ADEX subtype. Puleo et al. suggested that ADEX was likely an artifact resulting from contamination with transcripts from normal pancreatic acinar cells, rather than representing a true tumor-specific subtype.

The Cancer Genome Atlas (TCGA) Research Network performed an integrated genomic analysis of mRNA, miRNA, lncRNA, and DNA methylation profiles from 150 PDAC samples. They were able to identify two robust molecular subtypes: SNF-1 and SNF-2, based on Similarity Network Fusion (SNF) clustering [59]. The SNF-1 subtype corresponded to the basal-like subtype described by Moffitt et al., the squamous subtype in Bailey et al.’s classification, and the quasi-mesenchymal (QM) subtype in Collisson et al.’s study. This group was characterized by the expression of basal markers and was associated with poor prognosis. In contrast, the SNF-2 subtype aligned with the classical subtypes identified by both Moffitt et al. and Collisson et al., as well as the pancreatic progenitor subtype described by Bailey et al. SNF-2 tumors showed expression of differentiated ductal markers and were associated with more favorable clinical outcomes. Other subtypes proposed in earlier classifications, such as ADEX and immunogenic, were found to have low neoplastic cellularity in the TCGA analysis. This suggests that their molecular signatures may have been heavily influenced by stromal or normal pancreatic tissue contamination, rather than representing true tumor-intrinsic profiles [59].

Recently, Chan-Seng-Yue et al. examined 314 primary and metastatic PDACs through comprehensive whole-genome and transcriptome analysis of purified tumor cells. The authors identified five distinct molecular subtypes: basal-like A and B (related to the previously described basal-like subtype), hybrid, and classical A and B (corresponding to the classical subtype). Patients with basal-like B and hybrid tumors were more often diagnosed with resectable disease, whereas those with basal-like A tumors typically presented with advanced disease and exhibited the poorest response to gemcitabine-based chemotherapy and FOLFIRINOX. These subtypes are linked to specific genomic alterations: classical A and B tumors showed frequent *GATA6* amplification and complete loss of *SMAD4*, while basal-like A and B tumors were characterized by complete loss of *CDKN2A* and a higher incidence of *TP53* mutations. The hybrid subtype, marked by the presence of multiple expression signatures, did not align consistently with previously established classification systems. Importantly, single-cell analysis revealed that basal-like and classical subtype cells can coexist within the same tumor, underscoring the significant intratumoral molecular heterogeneity in PDAC [60].

Consensus has largely formed around two primary PDAC transcriptional subtypes—classical and basal-like—while leaving room for further subclassification. Notably, different subtypes can coexist within individual tumors. For example, Hwang et al. identified a treatment-enriched subtype through single-nucleus RNA sequencing and whole-transcriptome digital spatial profiling of 43 primary PDACs (18 untreated and 25 treated). They also discovered enrichment of a distinct neural-like progenitor (NRP) malignant cell program in residual tumors after chemoradiation therapy. NRP cells were associated with treatment resistance and poor survival, driven by upregulation of genes involved in inhibiting cell death and chemotherapy resistance (such as *ABCB1*, *BCL2*, *PDGFD*, and *SPP1*), neuronal migration and axonal guidance (including *SEMA3E*, *RELN*, and *SEMA5A*), and increased metastatic potential (NFIB) [61]. For a comprehensive overview of PDAC molecular subtypes, see Table 1.

## 5. Therapeutic Implications of Genetic Alteration in PDAC

### 5.1. Targeted Therapies

#### Targeting KRAS Mutations in PDAC

Gemcitabine/nab-paclitaxel and modified FOLFIRINOX are the National Comprehensive Cancer Network (NCCN)-recommended treatments for patients with locally advanced or metastatic PDAC, but their effectiveness remains limited. Despite numerous efforts, targeting the most common molecular alterations in PDAC has proven challenging [62].

KRAS mutations occur in over 90% of PDAC cases, yet the *KRAS^G12C^* mutation—which accounts for only about 1% of PDAC patients—has been the first KRAS mutant to be effectively targeted by a specific small-molecule inhibitor. Sotorasib irreversibly binds to a cryptic pocket in *KRAS^G12C^*, locking the protein in an inactive state and thereby inhibiting tumor growth [63]. Clinical trials have shown significant radiographic responses to sotorasib in *KRAS^G12C^*-mutated lung adenocarcinoma [64], leading to its approval by the U.S. Food and Drug Administration (FDA) as the first *RAS* inhibitor [65]. In a phase 1–2 clinical trial involving 38 patients with previously treated *KRAS^G12C^*-mutated advanced pancreatic cancer, sotorasib demonstrated promising results: eight patients achieved an objective response, with a median progression-free survival of 4.0 months and a median overall survival of 6.9 months [66]. Another *KRAS^G12C^* inhibitor, adagrasib, has also shown efficacy in preclinical models and produced radiographic responses in *KRAS^G12C^*-mutated lung and colon adenocarcinomas. The KRYSTAL-1 study demonstrated a clinical response to adagrasib in 7 of 21 (33%) patients with *KRAS^G12C^*-mutated PDAC [67]. Meanwhile, ASP3082 and MRTX1133 are emerging inhibitors that specifically target *KRAS^G12D^*—the most common KRAS mutation in PDAC—and are currently in preclinical development [68,69]. Given the high prevalence of *KRAS^G12D^* mutations in PDAC, these agents hold substantial promises for improving treatment outcomes. Daraxonrasib (RMC-6236) is an investigational pan-*RAS* inhibitor that has shown early activity and a manageable safety profile in patients with *RAS*-mutant PDAC. A phase 3 trial of this agent is currently underway [70], targeting non-*KRAS* driver mutations in PDAC.

Approximately 10% of PDACs do not harbor *KRAS* mutations [71], but instead feature a range of potentially druggable non-*KRAS* driver alterations. Among these, activating *BRAF* mutations occur in about 2% of PDACs, most commonly due to in-frame deletions or the V600E point mutation (*BRAFV600E*), which result in constitutive activation of the MAPK pathway [71]. In 2022, the FDA approved dabrafenib in combination with trametinib for the treatment of unresectable or metastatic solid tumors harboring a *BRAF V600E* mutation [72].

A recent study demonstrated that *MEK* inhibitors such as cobimetinib and trametinib were effective in regressing gemcitabine-resistant PDAC in patient-derived orthotopic xenograft (PDOX) models [73]. Based on these findings, a phase 2 multicenter clinical trial is underway to evaluate the combination of *BRAF* and *MEK* inhibitors (encorafenib and binimetinib, respectively) in patients with advanced *BRAFV600E*-mutated PDAC [73].

Neurotrophic receptor tyrosine kinase (*NTRK*) gene fusions, which drive mitogenic signaling in the central nervous system [74,75], are present in approximately 0.3% of PDACs [76]. These fusions arise from chromosomal rearrangements that result in the expression of chimeric tropomyosin receptor kinases. In a multicenter study of advanced cancers with *NTRK* fusions, including PDAC, 75% of patients responded to treatment with the TRK inhibitor larotrectinib, and 71% of those responses were sustained after one year [77]. These results led to the regulatory approval of larotrectinib for the treatment of advanced solid tumors with *NTRK* fusions. Similarly, entrectinib is approved for the treatment of solid tumors with *NTRK* fusions, irrespective of cancer type [78].

Activating neuregulin 1 (*NRG1*) fusions, identified in approximately 0.13–0.5% of pancreatic ductal adenocarcinomas (PDACs) [76,79], represent a promising therapeutic target. *NRG1* acts as a ligand for *ERBB3* and *ERBB4* receptors, and its fusion-driven overexpression promotes tumorigenesis through hyperactivation of *ERBB* signaling. This pathway is particularly relevant in *NRG1* fusion–positive, *KRAS* wild-type PDAC [80]. Reflecting this, the FDA recently approved zenocutuzumab-zbco for patients with advanced or metastatic PDAC harboring an *NRG1* gene fusion [81].

In a similar context, *RET* (rearranged during transfection) gene fusions, though rare, are clinically significant, occurring in approximately 0.6% of PDAC cases [82,83]. These fusions generate constitutively active *RET* receptor tyrosine kinases that drive cell proliferation and survival. Tumors with *RET* fusions often lack other canonical driver mutations, rendering them particularly sensitive to targeted therapy. Accordingly, the FDA has approved selpercatinib, a selective *RET* inhibitor, for adults with advanced or metastatic solid tumors harboring *RET* fusions, including PDAC [84].

### 5.2. DNA Repair Pathway in PDAC

Microsatellite instability (MSI) or deficient mismatch repair (dMMR) is observed in a small subset of PDACs (approximately 1–2%) [85] and is associated with a high tumor mutational burden. Tumors with MSI/dMMR are more likely to respond to immune checkpoint blockade. Reflecting this, the FDA has approved pembrolizumab, a PD-1 inhibitor, for the treatment of patients with advanced or metastatic MSI-high or dMMR solid tumors, including pancreatic cancer, providing a targeted immunotherapy option for this molecularly defined subgroup [86].

Similarly, defects in DNA repair due to germline *BRCA1* or *BRCA2* mutations, which are present in a subset of PDACs [87], create another actionable vulnerability. These tumors may be particularly sensitive to therapies that exploit deficiencies in DNA repair, such as platinum-based chemotherapies, which induce double-strand DNA breaks, or poly (ADP-ribose) polymerase (PARP) inhibitors, which block the repair of these lesions [1,88]. The POLO trial demonstrated that the PARP inhibitor olaparib was associated with longer progression-free survival in patients with germline *BRCA* mutation and metastatic pancreatic cancer who had previously responded to platinum-based chemotherapy [89]. In this randomized, placebo-controlled study, patients treated with olaparib experienced a median progression-free survival of 7.4 months, compared to 3.8 months in the placebo group. These findings led to FDA approval of olaparib as maintenance therapy for platinum-sensitive metastatic PDAC in patients with germline *BRCA* mutations [89].

In addition to *BRCA1/2*, other key regulators of DNA damage response, such as *ATM* and *ATR*, may serve as therapeutic targets. These genes encode members of the phosphatidylinositol 3-kinase-like kinase (PIKK) family, which play essential roles in DNA repair signaling. Tumors with *ATM* mutations have shown sensitivity to combined *ATR* and PARP inhibition, whereas PARP inhibitor monotherapy alone has demonstrated limited and short-lived activity in this context [90]. As a result, several early-phase clinical trials are currently underway to evaluate *ATR* inhibitors, both alone and in combination with cytotoxic chemotherapy or PARP inhibitors. For a summary of common genetic alterations in PDAC and corresponding targeted therapies, see Table 2.

### 5.3. Tumor Suppressor Pathways

*TP53* is the most commonly inactivated tumor suppressor gene in PDAC. Emerging therapeutic strategies are exploring ways to restore or modulate the *TP53* pathway. One such approach involves zinc chelators like COTI-2, which may stabilize mutant p53, promote proper protein folding, and prevent the aggregation of dysfunctional p53 [91]. Another strategy targets Mouse double minute 2 homolog (*MDM2*), a negative regulator of p53 that promotes its degradation through direct binding and ubiquitin-mediated mechanisms [92]. Inhibiting *MDM2* may help restore p53 activity in tumors with wild-type *TP53*. Another pathway of interest in PDAC is the transforming growth factor-beta (*TGF-β*)/*SMAD4* signaling cascade. This pathway plays a dual role in tumorigenesis, but in advanced stages, it promotes tumor invasion and metastasis. Inhibitors of *TGF-β*, such as trabedersen and galunisertib, have been shown to reduce tumor progression and metastatic potential in preclinical animal models [93]. *CDKN2A*, another frequently altered tumor suppressor gene in PDAC, encodes the proteins p16 and p19, which regulate cell cycle progression at the G1/S checkpoint through CDK4/6 inhibition [94]. Loss of *CDKN2A* function leads to unchecked cell proliferation. CDK4/6 inhibitors not only restore cell cycle control but have also been shown to impair DNA repair pathways and enhance the sensitivity of PDAC cells to PARP inhibitors [87]. These agents have demonstrated efficacy in preclinical PDAC models, and several related clinical trials are currently ongoing [94].

### 5.4. Other Oncogenic Pathways

The Wnt signaling pathway plays a key role in regulating cell differentiation, proliferation, and epithelial-to-mesenchymal transition [94]. Zhang et al. demonstrated that ligand-mediated activation of the Wnt/β-catenin pathway is essential for both the initiation and progression of PDAC [95]. In preclinical models, the monoclonal antibody vantictumab, which blocks Wnt signaling, was shown to reduce cancer stem cell frequency and enhance the efficacy of chemotherapy [96]. The JAK/STAT signaling pathway is also critically involved in tumor progression across several cancer types. In PDAC, elevated expression of *JAK* and activation of the *IL-6R/JAK/STAT* axis have been associated with poorer prognosis in patients with resectable disease [97]. Inhibition of *STAT3* has been found to enhance chemosensitivity and delay tumor progression [98]. Although *JAK* inhibitors such as itacitinib and momelotinib demonstrated acceptable safety profiles and some clinical activity in trials, they failed to produce a significant survival benefit when compared to chemotherapy alone [94]. Another pathway implicated in PDAC progression is Notch signaling, which is frequently upregulated and contributes to tumorigenesis. Through crosstalk with the Hedgehog, *KRAS*, and NF-κB pathways, Notch signaling promotes tumor cell proliferation and differentiation by modulating gene transcription [94]. As the pathway is activated by γ-secretase, inhibitors targeting this enzyme were developed as potential therapies. However, clinical trials were discontinued due to intolerable toxicity. Additional agents targeting Notch2 and Notch3 receptors have also been explored, but results to date have been suboptimal [99,100].

### 5.5. Stromal Targets and Tumor Microenvironment

PDAC is characterized by a dense fibrous stroma, resulting from the proliferation of fibrous tissue and alterations in the extracellular matrix (ECM). The stromal elements in PDAC play a critical role in regulating tumor growth, vascularization, immune response, drug delivery, and metastasis [101]. The ECM consists of a complex network of structural proteins, adaptor molecules, proteoglycans, and enzymes that contribute to the rigidity and resistance of the tumor microenvironment. One key ECM component is hyaluronic acid (HA), which increases interstitial pressure within the tumor, leading to vascular collapse and reduced perfusion [102]. This limits the accessibility of chemotherapeutic agents to cancer cells. Therapeutic approaches targeting this barrier, such as recombinant human hyaluronidase (PEGPH20), have been explored, but clinical trials have yielded disappointing results [103]. Matrix metalloproteinases (MMPs) are another group of stromal modifiers that degrade ECM components and the basement membrane, thereby facilitating tumor invasion, angiogenesis, and metastasis. Marimastat, an MMP inhibitor, demonstrated safety and biological activity in early-phase studies but failed to provide a survival benefit when combined with gemcitabine in clinical trials [94].

### 5.6. Therapeutic Implication of PDAC Molecular Subtype

Molecular subtyping of advanced PDAC provides valuable insights into the determinants of chemotherapy response. The recent COMPASS trial evaluated this by performing tumor biopsies followed by RNA sequencing in 195 patients. Tumors were classified into two major subtypes: classical (80%) and basal-like (20%) [104]. Patients with the classical subtype showed significantly better responses to first-line 5-fluorouracil–based chemotherapy regimens, while those with the basal-like subtype exhibited reduced chemo-responsiveness [104]. Ongoing validation studies aim to further refine the clinical utility of PDAC subtyping in guiding personalized treatment strategies. 

## 6. Conclusions

Over the past few decades, genomic research has greatly advanced our understanding of the complex molecular landscape of PDAC. Although many insights have yet to be fully translated into clinical practice, the identification of key genetic alterations and molecular subtypes offers opportunities to optimize existing therapies and develop novel, targeted treatments. Frequent mutations have been identified in critical genes such as *KRAS*, *TP53*, *SMAD4/DPC4*, and *CDKN2A*, along with numerous less common alterations. Clear genotype–phenotype correlations across histologic subtypes and precursor lesions, combined with transcriptomic analyses, have further informed the development of targeted therapies. Particularly encouraging are recent clinical trials of *KRAS*-specific inhibitors and the FDA approval of PARP inhibitors, both representing meaningful strides forward in PDAC treatment.

## Figures and Tables

**Table 1:** Molecular subtypes of PDAC.

Reference, Year	Subtypes	Relevant Clinical Information
Collisson et al., 2011 [55]	Classical	Best survival, more sensitive to erlotinib
	Quasi-mesenchymal	Poorest prognosis, more sensitive to gemcitabine
	Exocrine-like	High expression of genes related to digestive enzymes
Moffitt et al., 2015 [56]	Classical	Resemble the classical group from Collisson et al.
	Basal-like	Poor prognosis
Bailey et al., 2016 [57]	Squamous	Frequent TP53 mutations, worse survival
	Pancreatic progenitor	Association with transcriptional factors and metabolic pathways
	Aberrantly differentiated endocrine exocrine (ADEX)	Upregulation of genes involved in KRAS activation and endocrine and exocrine differentiation
	Immunogenic	Upregulated immune network
Cancer Genome Atlas Research Network, 2017 [59]	SNF-1	Poor prognosis
	SNF-2	Better prognosis
Puleo et al., 2018 [58]	Pure basal-like	
	Stroma-activated	
	Desmoplastic	
	Pure classical	
	Immune classical based	
Chan-Seng-Yue, 2020 [60]	Basal-like A	Advanced disease, worst response to gemcitabine and FOLFIRINOX
	Basal-Like B	Resectable disease
	Hybrid	Resectable disease
	Classical A and B	GATA6 amplification, complete SMAD4 loss

**Table 2:** Common genetic alterations in PDAC.

High-Frequency Alterations	FDA-Approved Targeted Therapy (Indication)
*CDKN2A*/*p16*	Sotorasib—Adagrasib (G12C variant)
*KRAS*	
*TP53*	
*SMAD4*/*DPC4*	
Low frequency alterations	Targeted therapy
*AKT2*	
*BRAF*	Encorafenib (V600E variant)
*BRCA1*/*2*	Olaparib (germline mutation)
*NRG1*	Zenocutuzumab (gene fusion)
*NTRK*	Larotrectinib (gene fusion)
*RET*	Selpercatinib (gene fusion)
*STK11*/*LKB1*	
Microsatellite Instability	Pembrolizumab (MSI-H or dMMR)
